# Differential response of skeletal muscles to mTORC1 signaling during atrophy and hypertrophy

**DOI:** 10.1186/2044-5040-3-6

**Published:** 2013-03-06

**Authors:** C Florian Bentzinger, Shuo Lin, Klaas Romanino, Perrine Castets, Maitea Guridi, Serge Summermatter, Christoph Handschin, Lionel A Tintignac, Michael N Hall, Markus A Rüegg

**Affiliations:** 1 Biozentrum, University of Basel, Basel, CH-4056, Switzerland; 2 Neuromuscular Research Center, Department of Biomedicine, University of Basel, Basel, CH-4056, Switzerland; 3 INRA, UMR866, Université Montpellier 1, Université Montpellier 2, Montpellier, France

**Keywords:** Skeletal muscle, Hypertrophy, Atrophy, Mammalian target of rapamycin complex 1 (mTORC1), Raptor, Tuberous sclerosis complex (TSC), PKB/Akt, FoxO, MuRF1, Atrogin-1/MAFbx

## Abstract

**Background:**

Skeletal muscle mass is determined by the balance between protein synthesis and degradation. Mammalian target of rapamycin complex 1 (mTORC1) is a master regulator of protein translation and has been implicated in the control of muscle mass. Inactivation of mTORC1 by skeletal muscle-specific deletion of its obligatory component raptor results in smaller muscles and a lethal dystrophy. Moreover, raptor-deficient muscles are less oxidative through changes in the expression PGC-1α, a critical determinant of mitochondrial biogenesis. These results suggest that activation of mTORC1 might be beneficial to skeletal muscle by providing resistance to muscle atrophy and increasing oxidative function. Here, we tested this hypothesis by deletion of the mTORC1 inhibitor tuberous sclerosis complex (TSC) in muscle fibers.

**Method:**

Skeletal muscles of mice with an acute or a permanent deletion of raptor or TSC1 were examined using histological, biochemical and molecular biological methods. Response of the muscles to changes in mechanical load and nerve input was investigated by ablation of synergistic muscles or by denervation .

**Results:**

Genetic deletion or knockdown of raptor, causing inactivation of mTORC1, was sufficient to prevent muscle growth and enhance muscle atrophy. Conversely, short-term activation of mTORC1 by knockdown of TSC induced muscle fiber hypertrophy and atrophy-resistance upon denervation, in both fast *tibialis anterior* (TA) and slow *soleus* muscles. Surprisingly, however, sustained activation of mTORC1 by genetic deletion of *Tsc1* caused muscle atrophy in all but *soleus* muscles. In contrast, oxidative capacity was increased in all muscles examined. Consistently, TSC1-deficient *soleus* muscle was atrophy-resistant whereas TA underwent normal atrophy upon denervation. Moreover, upon overloading, *plantaris* muscle did not display enhanced hypertrophy compared to controls. Biochemical analysis indicated that the atrophy response of muscles was based on the suppressed phosphorylation of PKB/Akt via feedback inhibition by mTORC1 and subsequent increased expression of the E3 ubiquitin ligases *MuRF1* and *atrogin-1/MAFbx*. In contrast, expression of both E3 ligases was not increased in *soleus* muscle suggesting the presence of compensatory mechanisms in this muscle.

**Conclusions:**

Our study shows that the mTORC1- and the PKB/Akt-FoxO pathways are tightly interconnected and differentially regulated depending on the muscle type. These results indicate that long-term activation of the mTORC1 signaling axis is not a therapeutic option to promote muscle growth because of its strong feedback induction of the E3 ubiquitin ligases involved in protein degradation.

## Background

Skeletal muscle is the largest organ, accounting for 30 to 40% of the total body weight. Muscle tissue is highly plastic and adapts its size to physical demand. For example, increase in load causes hypertrophy whereas unloading causes atrophy. Importantly, muscle atrophy and subsequent wasting are also hallmarks of pathology in muscular dystrophies or in cachexia, the latter being a secondary consequence of a primary disease (for example, AIDS, cancer or sepsis). Several lines of evidence indicate that muscle mass is controlled by the balance between protein synthesis and protein degradation [1,2]. In skeletal muscle, protein synthesis can be induced by IGF1 (insulin-like growth factor-1), which in turn, activates PI3K (phosphatidylinositol 3-kinase) and PKB (protein kinase B; also called Akt). Activated PKB/Akt inhibits the protein complex TSC1-TSC2 (tuberous sclerosis complex), which inactivates the small GTPase protein Rheb (Ras homolog enriched in brain). Rheb activates mammalian target of rapamycin complex 1 (mTORC1), which causes an increase in protein translation by phosphorylating its two best characterized targets S6K (p70S6 kinase) and 4EBP (eIF-4E-binding protein). This IGF1-PI3K-PKB/Akt-mTOR signaling pathway controls protein synthesis and cell size in several tissues [3,4].

Activation of PKB/Akt also negatively regulates protein degradation by phosphorylating the FoxO (Forkhead box O) transcription factors. Protein degradation is mainly carried out by enzymes of the ubiquitin-proteasomal and autophagosomal-lysosomal pathways [5,6]. Dephosphorylated FoxOs in the nuclei promote the expression of the two E3 ubiquitin ligases *atrogin-1/MAFbx* and *MuRF1*[7,8]. FoxOs have also been described to drive expression of autophagy-related genes [6,9]. The function of active PKB/Akt to simultaneously stimulate protein synthesis and inhibit protein degradation may explain the profound hypertrophic effect of constitutively active PKB/Akt [10,11].

mTOR belongs to the PI3/PI4-kinase family; it is highly conserved from yeast to human and assembles into two structurally and functionally distinct multiprotein complexes, called mTORC1 and mTORC2 [12,13]. An essential component of mTORC1 is the protein raptor (regulatory-associated protein of mTOR), whereas rictor (rapamycin-insensitive companion of mTOR) is an essential subunit of mTORC2 [3,4]. Most functions of mTORC1 are acutely inhibited by the immunosuppressant rapamycin, whereas mTORC2 is only repressed by long-term application of rapamycin [14]. In skeletal muscle, the function of mTORC2 seems to not be essential because mice deficient for rictor have no overt phenotype [15,16]. In contrast, mTORC1 participates in the control of muscle size. For example, rapamycin prevents IGF1-induced growth of myotubes [17], inhibits compensatory hypertrophy in rat skeletal muscle [5] and blocks the growth-stimulating activity of clenbuterol [18]. Moreover, transgenic overexpression of TSC1 causes muscle atrophy in mice [19], while acute overexpression of Rheb induces muscle hypertrophy [20]. Finally, mice deficient for S6K1 show a reduction of muscle fiber size and a blunted response to IGF1 [21].

In agreement with these findings, we recently showed that mice with a skeletal muscle-specific knockout for raptor (called RAmKO for raptor muscle knockout) have a reduced muscle mass and suffer from a progressive dystrophy, which causes their death at the age of four to six months [16]. Muscles of RAmKO mice also have a decreased oxidative capacity, which can be restored by transgenic expression of PGC-1α [22]. In addition, RAmKO mice show sustained activation of PKB/Akt because of relieved feedback inhibition onto IRS1 (insulin receptor substrate-1) by the diminished activation of S6K [16].

Here we investigated the contribution of mTORC1 to muscle atrophy and hypertrophy by targeting *rptor* (the gene encoding raptor) or *Tsc1* (encoding TSC1) specifically in mouse skeletal muscle. We show that deletion of *rptor* prevents muscle hypertrophy and enhances muscle atrophy. Surprisingly, sustained activation of mTORC1 by the genetic deletion of *Tsc1* does not induce hypertrophy but rather causes atrophy in all but *soleus* muscles. While the TSC1-deficient, hypertrophic *soleus* muscle is also resistant to denervation-induced atrophy, *tibialis anterior* (TA) muscle atrophies like controls. Biochemical characterization shows that regulation of the two E3 ligases atrogin-1/MAFbx and MuRF1 differs between TA and *soleus* muscles. Furthermore, we demonstrate that all muscles show an increase in their oxidative capacity upon mTORC1 activation. In summary, we demonstrate that the oxidative capacity in all skeletal muscles is controlled by mTORC1, whereas the effect of sustained activation of mTORC1 on muscle size differs between muscles. Hence, our studies decipher a mechanism of biological robustness that balances the two major metabolic pathways involved in the control of skeletal muscle mass.

## Methods

### Mice

Mice were maintained in a conventional facility with a fixed dark–light cycle. Studies were carried out according to criteria outlined for the care and use of laboratory animals and with approval of the Swiss authorities. RAmKO mice were generated and genotyped as described before [16]. Floxed *Tsc1* mice [23] were obtained from The Jackson Laboratory (Bar Harbor, Maine, USA) and mated with mice expressing Cre recombinase under the human skeletal actin (HSA) promoter [24]. Genotyping for the conditional *Tsc1* allele was performed as described [23]. TSC-RAmKO mice were generated by intercrossing mice carrying floxed *rptor* and *Tsc1* alleles. Mice homozygous for both floxed alleles were mated with double heterozygotes, which also carried the *HSA-Cre* transgene. Except for overloading experiments and Western blot analysis, only male TSCmKO mice were used. Both genders were used in RAmKO and TSC-RAmKO mice. All procedures were performed in accordance with the Swiss regulations for animal experimentation and they were approved by the veterinary commission of the Canton Basel-Stadt.

### Rapamycin treatment of mice

Rapamycin treatment began three days before the mice were challenged with functional overload (FO) or electroporation and continued until mice were sacrificed. Rapamycin (LC Laboratories, Woburn, MA, USA), dissolved in saline containing 2% carboxymethylcellulose (Sigma-Aldrich, St. Louis, MO, USA), was delivered once daily by i.p. (intraperitoneal) injection at a dose of 1.5 mg/kg [5].

### shRNA constructs

The methods to construct plasmids encoding shRNA and the sequences of the *Cd4* control shRNA and the NLS-GFP construct have been described elsewhere [25]. The murine 19 nucleotide target sequences correspond to: GTT GAT GCG TAA CCT TCT G (*Tsc2*), GAT GGA CAC TGA TGT TGT G (*Tsc1*) and GAA TTT TGC TGA TTT GGA A (*rptor*).

### Tissue culture, transfections and shRNA efficiency

Adenoviruses encoding shRNA against *Tsc2* and *Cd4* were created by cloning the respective shRNA sequence and H1 promoter from pSuper into pAd-DEST (Life Technologies Europe B.V., Zug, Switzerland). To test the efficiency of the *Tsc2* shRNA, C2C12 myoblasts, cultured under standard conditions, were transfected with the *Tsc2* and *Cd4* shRNA viruses. The efficiency of the *rptor* shRNA was tested by co-transfection with an expression plasmid encoding HA-tagged raptor into COS7 cells using Lipofectamine 2000 (Life Technologies). For PGC1β overexpression and knockdown, myoblasts were permitted to fuse into multinucleated myotubes for 48 hr and cells were infected with adenovirus preparations for an additional 48 hr. Adenoviruses (Ad-GFP, Ad-PGC1β, Ad-scrambled or Ad-siPGC1β) were kindly provided by BM Spiegelman (Harvard University, Boston, MA, USA).

### Electroporation of muscle

Plasmids encoding shRNA constructs were electroporated into muscle fibers as described before [25]. Briefly, *soleus* or TA muscle of anesthetized mice was exposed and injected with 10 to 30 μl of a mixture containing the respective shRNA plasmid and a plasmid coding for NLS-GFP (2 mg/ml of each construct). The fascia and the skin were sutured and the electroporation was performed using an ECM 830 electroporation system (BTX Instruments Division, Harvard Apparatus Inc., Holliston, MA, USA). Eight pulses lasting 20 ms with the frequency of 1 Hz and the voltage set to 180 V/cm were applied. Mice were analyzed four to six weeks after electroporation.

### Denervation, nerve crush and overloading

Mice were anesthetized with ketamine (111 mg/kg) and xylazine (22 mg/kg) by intra-peritoneal injection and surgery was performed under aseptic conditions. For denervation, a segment (approximately 5 mm) of the sciatic nerve at the mid-thigh level was excised [26]. To induce muscle re-growth, the nerve was crushed with No 5 Dupont forceps (Fine Science Tools GmbH, Heidelberg, Germany) for 10 seconds at mid-thigh [27]. To induce muscle hypertrophy, a functional overload of *plantaris* muscle was introduced by surgical removal of *soleus* and *gastrocnemius* muscles [28]. Surgery was performed on one leg only. The *plantaris* muscle of the contralateral leg served as control.

### Antibodies

The antibodies used were from the following sources: rabbit polyclonal antibodies directed to 4E-BP1 (Phas-I) from Zymed (Life Technologies); those recognizing Phospho-4E-BP1 (Ser65), PKB/Akt, mTOR, S6 Ribosomal Protein or Phospho-S6 Ribosomal Protein (Ser235/236) were all from Cell Signaling Technology Inc. (Danvers, MA, USA); those against FoxO1a were from Abcam plc. (Cambridge, UK); those against TSC1 were from Bethyl Laboratories (Montgomery, TX, USA). Rabbit monoclonal antibodies directed against Phospho-Akt (Ser473), IRS-1, FoxO3a (75D8) and phospho-FoxO1(Thr24)/FoxO3a(Thr32) (#9466) were from Cell Signaling Technology Inc. Mouse monoclonal antibodies to α-actinin were purchased from Sigma and antibodies against HA from Covance Inc. (Geneva, Switzerland). Rat monoclonal antibodies directed to the Laminin B2 Chain (MAB1914) were from Chemicon and sold by Millipore AG (Zug, Switzerland). The TSC2 antibodies used were described elsewhere [29]. Mouse monoclonal antibodies against myosin heavy chain: slow (A4.840), IIa/IIx (A4.74) and IIb (BF-F3) were purchased from The Developmental Studies Hybridoma Bank (University of Iowa, Iowa City, Iowa, USA). Antibodies to puromycin [30] were a kind gift of Dr. Philippe Pierre (CIML Parc Scientifique de Luminy, Marseille, France).

### Histology and immunohistochemistry

Muscles frozen in liquid nitrogen-cooled isopentane were cut into 12 μm cross-sections. Cross-sections were fixed with 2% paraformaldehyde (PFA) and permeabilized with 1% Triton/PBS for 5 minutes, washed with 100 mM glycine/PBS for 15 minutes, blocked with 1% BSA/PBS for 30 minutes, and incubated with the primary antibody overnight at 4°C. Samples were subsequently washed three times for 10 minutes each with 1% BSA/PBS and stained with the appropriate fluorescence labeled secondary antibodies for 1 hr at room temperature. After washing with PBS, samples were mounted with Citifluor (Citifluor Ltd. London, UK). General histology on cross-sections was performed using hematoxylin and eosin (H&E; Merck, Zug, Switzerland). NADH-TR (Nicotinamide adenine dinucleotide hydrogen-tetrazolium reductase) staining was done as described [31]. Methods of SDH and COX staining were described elsewhere [22]. Samples were dehydrated and mounted with DePeX mounting medium (Gurr, BDH, VWR International GmbH, Dietikon, Switzerland).

### *In vivo* protein synthesis

Protein synthesis was measured using the surface sensing of translation (SUnSET) method [30] by i.p. injection of 0.040 μmol/g puromycin dissolved in 100 μl of PBS. Mice were sacrificed 30 minutes later and muscles were snap-frozen in liquid nitrogen. Muscles were lysed as described below and proteins were separated on 8 to 16% SDS-PAGE (Bio-Rad Laboratories AG, Cressier, Switzerland). After transfer to polyvinyl difluoride membranes and blocking of free binding sites with 5% milk powder in Tris-buffered saline with 0.1% Tween 20 (TBST), the mouse IgG2a monoclonal anti-puromycin antibody (clone 12D10; 1:5,000) was incubated for 1 hr at room temperature. After incubation with the appropriate HRP-coupled secondary antibody, blots were developed using enhanced chemiluminescence reagent. Coomassie Blue staining was used to verify equal loading.

### Tissue homogenization, SDS-PAGE and Western blot

Muscles frozen in liquid nitrogen were powdered on dry ice and lysed in cold RIPA buffer supplemented with 1% Triton-X, 10% glycerol, protease inhibitor cocktail tablets (Roche Diagnostics AG, Rotkreuz, Switzerland), and phosphatase inhibitor cocktail I and II (Sigma). Cell lysates were incubated on ice for 2 hr, sonicated two times for 15 s and centrifuged at 13,600 g for 30 minutes at 4°C. Cleared lysates were then used to determine total protein levels (BCA Protein Assay, Pierce, Rockford, IL, USA). After dilution with sample buffer, equal protein amounts were loaded onto SDS gels.

### Real-time PCR

Total RNA was isolated (SV Total RNA Isolation System, Promega AG, Dübendorf, Switzerland) from *soleus* muscles. RNA concentrations were adjusted between samples and reverse transcription was carried out using a mixture of oligodT and random hexamer primers (iScript cDNA Synthesis Kit, Bio-Rad Laboratories AG). Sybr Green, real-time PCR analysis (Power SYBR Green Master Mix, Life Technologies) was performed using the ABI Prism 7000 Sequence Detector. Expression levels for each gene of interest were normalized to the expression of the housekeeping protein β-actin. The following primers were used: *β-actin* sense primer: 5' CAG CTT CTT TGC AGC TCC TT, antisense primer: 5' GCA GCG ATA TCG TCA TCC A; *atrogin-1/MAFbx* sense primer: 5' CTC TGT ACC ATG CCG TTC CT, antisense primer: 5' GGC TGC TGA ACA GAT TCT CC; *MuRF-1* sense primer: 5^′^ ACC TGC TGG TGG AAA ACA TC, antisense primer: 5^′^ AGG AGC AAG TAG GCA CCT CA; *Pgc1α* sense primer: 5’ TGA TGT GAA TGA CTT GGA TAC AGA CA, antisense primer: 5’ GCT CAT TGT TGT ACT GGT TGG ATA TG; *Pgc1β* sense primer: 5' GGC AGG TTC AAC CCC GA, antisense primer: 5' CTT GCT AAC ATC ACA GAG GAT ATC TTG. Quantification of mitochondrial DNA copy numbers was done as described [22].

### Quantifications and statistics

For muscle fiber size quantification, muscle cross-sections were stained either for laminin-γ1 or fluorescence labeled wheat-germ agglutinin. Images were acquired using a Leica DM5000B fluorescence microscope with 10x objective, a digital camera (F-View; Soft Imaging System, Olympus Soft Imaging Solutions GmbH, Münster, Germany), and analySIS software (Soft Imaging System). Images of the entire *soleus* or *tibialis anterior* (TA) muscles were aligned with Adobe PhotoShop (Adobe Systems Incorporated, San Jose, CA, USA). The minimum distance of parallel tangents at opposing particle borders (minimal feret’s diameter) and cross-section area (CSA) were measured with analySIS software as described [32]. Data are expressed as mean ± SEM. For statistical comparison of two conditions, the Student’s *t*- test was used. The level of significance is indicated as follows: *** *P* <0.001, ** *P* <0.01, * *P* <0.05.

## Results

### Acute changes in mTORC1 activity affect muscle fiber size

**Figure 1:**
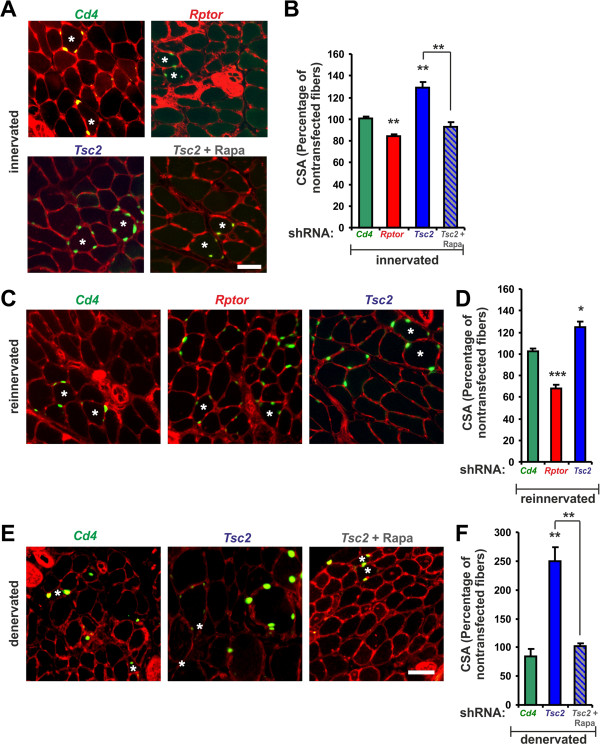
**Acute perturbation of mTORC1 affects muscle fiber size.***Soleus* muscle was electroporated with plasmids encoding shRNA directed to transcripts encoding CD4 (*Cd4*), raptor (*Rptor*) or TSC2 (*Tsc2*). Plasmids encoding NLS-GFP were co-electroporated to label transfected fibers. After four to six weeks, muscle fiber size was determined by staining mid-belly cross-sections with Alexa-594-labeled wheat germ agglutinin (red). Transfected muscle fibers were identified by the expression of nuclear-localized GFP (green; white asterisks). The experimental paradigms used were innervated muscle (**A, B**), reinnervated muscle after nerve crush (**C, D**) and denervated muscle (**E, F**). Quantifications (**B, D, F**) of cross-sectional area (CSA) of muscle fibers in each paradigm are given relative to CSA of neighboring, GFP-negative, non-electroporated fibers. Electroporation of plasmids encoding shRNA to *Cd4* served as control. Scale bars (**A, C, E**) = 50 μm. Bars (**B, D, F**) represent mean ± SEM (N ≥3 mice and N ≥200 fibers were measured in each). In case of innervated muscles treated with shRNA to *Tsc2* and with rapamycin (*Tsc2* + Rapa) and denervated muscles electroporated with shRNA to *Cd4*, data represent mean ± SD (N = 2). *P*-values are ***P <0.001; **P <0.01; *P <0.05. Unless otherwise indicated, significance was determined compared to the control (shRNA to *Cd4*).

To evaluate the potential of mTORC1 in regulating muscle fiber size, we first tested the effect of mTORC1 inhibition or activation in normal weight-bearing muscles and in acute models of muscle hypertrophy and atrophy. To this end, we electroporated plasmids encoding an shRNA directed against *rptor* (to inactivate mTORC1) or *Tsc2* (to activate mTORC1) into muscle fibers of mouse *soleus* muscle using the methods described [25]. As a negative control, shRNA constructs directed against *Cd4* were used. To label targeted muscle fibers, a plasmid coding for nuclear-localized GFP (NLS-GFP) was co-electroporated with all shRNA constructs. Before electroporation into muscle, each shRNA construct was tested in tissue culture using either COS cells co-transfected with the corresponding expression plasmid (Additional file 1: Figure S1A) or by infecting myoblasts with adenovirus expressing the corresponding shRNA construct (Additional file 1: Figure S1B). Four to six weeks after electroporation, transfected muscle fibers were identified by their expression of NLS-GFP in myonuclei (Figure 1) and the size of GFP-positive fibers was compared with that of neighboring, non-transfected fibers. Knockdown of raptor resulted in a small but significant decrease in muscle fiber size, whereas knockdown of TSC2 resulted in a significant increase (Figure 1A, B). Consistent with the notion that TSC1/2 acts via mTORC1, rapamycin fully prevented the muscle hypertrophy observed in TSC2 knockdown fibers (Figure 1A, B). As expected, electroporation of shRNA constructs targeting *Tsc1* resulted in a hypertrophy response very similar to the *Tsc2* knockdown (Additional file 1: Figure S1C, D).

To test the role of mTORC1 in muscle plasticity, we crushed the sciatic nerve unilaterally immediately after electroporation, which causes a transient denervation-induced atrophy, followed by fiber re-innervation and re-growth to normal size [27,33,34]. Such “hypertrophy on recovery” (HOR) was significantly less in muscle fibers expressing shRNA to *rptor* and significantly higher in fibers expressing shRNA to *Tsc2* (Figure 1C, D). To test whether shRNA-targeting acted on the initial atrophy or on re-growth, we also examined electroporated muscle fibers in a pure denervation-induced atrophy paradigm. No difference between non-electroporated and electroporated fibers was detected in *Cd4* controls (Figure 1E, F). In contrast, muscle fibers expressing shRNA to *Tsc2* were much bigger than non-electroporated fibers and, like in innervated muscle, the effect of TSC2 knockdown was abrogated by rapamycin (Figure 1E, F). Similar results were obtained by electroporating *tibialis anterior* (TA) muscle (Additional file 1, Figure S1E, F). These results thus show that acute alteration of mTORC1 activity affects the response of both, the slow oxidative *soleus* and fast glycolytic TA muscles to growth-stimulating and atrophy-inducing conditions.

### Constitutive deletion of *Tsc1* in skeletal muscle fibers affects muscles differentially

**Figure 2:**
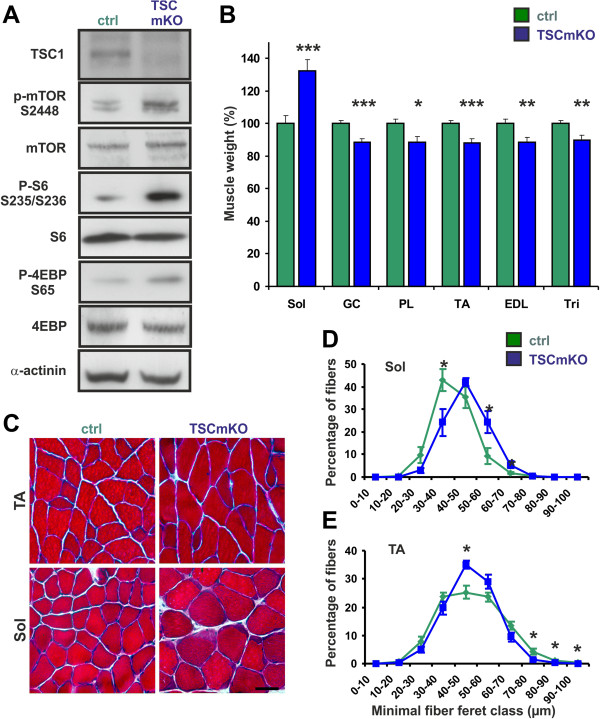
**Conditional inactivation of TSC1 in skeletal muscle.** (**A**) Western blot analysis of *soleus* muscle from 90-day-old control (ctrl) and TSCmKO mice using antibodies directed against the proteins indicated. α-actinin is used as a loading control. (**B**) Weight of *soleus* (Sol), *gastrocnemius* (GC), *plantaris* (PL), *tibialis anterior* (TA), *extensor digitorum longus* (EDL) and *triceps* (Tri) muscles of TSCmKO and littermate control (ctrl) mice. Weight is expressed as a percentage of the weight of the same muscle in control mice after normalization to the total body weight (N = 8 to 12 mice for each genotype). Data are mean ± SEM; ***P <0.001; **P <0.01; *P <0.05; Student’s *t*-test. (**C**) H&E staining of cross-sections from TA and *soleus* muscles of control and TSCmKO mice. Scale bar = 50 μm. (**D, E**) Fiber size distribution in *soleus* (**D**) and TA (**E**) muscles of 90-day-old TSCmKO and control mice (N = 4). More details of fiber size analysis are shown in Additional file 1: Figure S3 and in Additional file 1, Table S1. *P <0.05.

To examine whether sustained activation of mTORC1 would lead to the same effects observed in our electroporation paradigm, mice carrying floxed alleles for *Tsc1*[23] were crossed with mice that express Cre recombinase under the control of the muscle fiber-specific human skeletal actin (HSA) promoter [24]. Mice lacking TSC1 in skeletal muscle (herein called TSCmKO, for TSC muscle knockout) were born at the expected Mendelian ratio and, at birth, could not be visually distinguished from their littermate controls. Muscle extracts from TSCmKO mice were largely devoid of TSC1 (Figure 2A). Moreover, they showed the expected increase in phosphorylation of mTOR at the mTORC1-selective site Serine 2448 and of the mTORC1 targets S6 and 4EBP (Figure 2A; Table 1 for quantification). These data are similar to those obtained in other tissues where *Tsc1* or *Tsc2* were conditionally ablated [23,35-37].

**Table 1:** Quantification of Western blot analysis

	**TSCmKO**	**Ctrl**	**Ratio**	**Number of replicates**
**p-mTOR**^**S2448**^	19 ± 2***	10 ± 1	1.9	4
**mTOR**	31 ± 6	28 ± 5	1.1	4
**P-4E-BP1**^**S65**^	23 ± 5***	12 ± 3	1.9	4
**4E-BP1**	34 ± 6	39 ± 6	0.9	4
**P-S6**^**S235/S236**^	53 ± 1***	13 ± 6	4	4
**S6**	48 ± 11	42 ± 17	1.1	4
**IRS-1**	6 ± 2***	24 ± 1	0.3	4
**P-PKB/ Akt**^**S473**^	4 ± 2***	22 ± 3	0.2	4
**PKB/ Akt**	28 ± 7	22 ± 6	1.2	4
**FoxO1**	23 ± 5	18 ± 3	1.3	4
**FoxO3a**	16 ± 7	17 ± 5	0.9	4
**P-FoxO1**^**T24**^**/3a**^**T23**^	2 ± 7**/ 8 ± 3*	11 ± 2/ 20 ± 5	0.2/ 0.4	4
**P-PKB/ AktS**^**473 **^**(Den. TA)**	n.d.	n.d.	n.d.	3
**P-S6**^**S235/S236 **^**(Den. TA)**	47 ± 12***	5 ± 2	9.4	3
**P-PKB/ Akt**^**S473 **^**(Den. Sol)**	n.d.	n.d.	n.d.	3
**P-S6**^**S235/S236 **^**(Den. Sol)**	41 ± 10**	3 ± 5	13.7	3
	**RAmKO**	**Ctrl**	**ratio**	**number of replicates**
**P-PKB/ Akt**^**S473 **^**(Den. TA)**	12 ± 2	2 ± 1	6	3
**P-S6**^**S235/S236 **^**(Den. TA)**	n.d.	n.d.	n.d.	3
**P-PKB/ Akt**^**S473 **^**(Den. Sol)**	19 ± 3	3 ± 2	6.3	3
**P-S6**^**S235/S236 **^**(Den. Sol)**	n.d.	n.d.	n.d.	3

Proteins were extracted from *soleus* (Sol) and *tibialis anterior* (TA) muscles of 90-day-old TSCmKO, RAmKO or control (Ctrl) littermates. “Den.” denotes: denervated. “n.d” denotes: not detectable. The amount of total protein loaded onto the SDS-PAGE was adjusted and Western blots were additionally normalized to α-actinin levels. Numbers given represent average gray values ± SEM after subtraction of the background. “Ratio” represents the average gray value obtained from a knockout animal divided by the gray values from the control littermates. “Number of replicates” represents the number of knockout animals analyzed. The number of Ctrl littermates was always the same or higher than the values given. *P*-values were determined by Student’s *t*-test; * *P* <0.05, ** *P* <0.01, ***P <0.001.

Consistent with the activation of the mTORC1 targets and the role of mTORC1 in the control of protein translation, protein synthesis in EDL muscle of TSCmKO was increased (Additional file 1: Figure S2A). However, TSCmKO mice gained less weight than their control littermates. Starting from the age of five weeks, male TSCmKO mice were significantly lighter (Additional file 1: Figure S2B), whereas the weight difference in females did not reach significance (Additional file 1: Figure S2C). At least part of this weight difference was due to alteration in muscle mass as all but *soleus* muscles were significantly lighter than in control mice (Figure 2B). Thus, despite increased protein synthesis, all but soleus muscles are lighter in TSCmKO mice than in control mice.

To investigate the reason for these muscle-specific differences in weight, we focused on *soleus* and TA muscles in three-month-old mice. Hematoxylin & eosin (H&E) staining did not reveal any major alterations in either of the muscles (Figure 2C). The difference in the muscle weight was matched by changes in the muscle fiber size in *soleus* and TA muscle (Figure 2D, E). Detailed analysis of fiber types showed that both type I and type IIa fibers were larger in *soleus* muscle (Additional file 1: Figure S3; Additional file 1: Table S1). In TA muscle, the glycolytic type IIb fibers were significantly smaller whereas the oxidative type IIa/x fibers were not affected (Additional file 1: Figure S3, Additional file 1: Table S1). In summary, these data show that the response to the activation of mTORC1 differs between muscles and fiber types.

**Figure 3:**
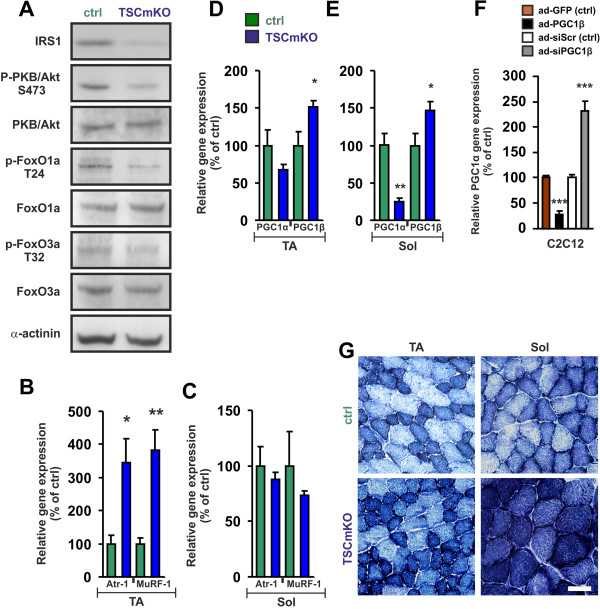
**mTORC1 activation affects the PKB/Akt and PGC1 pathways.** (**A**) Western blot analysis of *soleus* muscles from 90-day-old control (ctrl) and TSCmKO mice using antibodies directed against the proteins indicated. α-actinin is used as loading control. (**B, C**) Relative mRNA expression of *atrogin-1/MAFbx (*Atr-1*)* and *MuRF1* in TA and *soleus* muscles of TSCmKO and control mice. All values were normalized to the expression of *β-actin* and control muscles were set to 100% (TA: N ≥4 mice; Sol: N ≥5 mice). (**D, E**) Relative mRNA expression of *Pgc1α* and *Pgc1β* is shown in TA (**D**) and *soleus* (**E**) muscles of TSCmKO and control mice. All values are normalized to expression of *β-actin*. Relative expression in muscles from control littermates were set to 100%. TA: N ≥4; Sol: N ≥5. Note that levels of *Pgc1β* but not *Pgc1α* are up-regulated in TSCmKO mice. (**F**) Relative mRNA levels of *Pgc1α* in differentiated C2C12 cells that were infected with adenoviral vectors encoding GFP (ad-GFP), PGC1β (ad-PGC1β), shRNA to a scrambled sequence (ad-siScr) or shRNA to *Pgc1β* (ad-siPGC1β). Values are normalized to each control (ad-GFP and ad-siScr) and were set to 100% (N = 9). Note that expression of *Pgc1α* inversely correlates with *PGC1β* levels. Quantitative data (**B-F**) represent mean ± SEM. *P*-values are ***P <0.001; **P <0.01; *P <0.05; Student’s *t*-test. (**G**) NADH-TR staining of TA and *soleus* muscles of 90-day-old control and TSCmKO mice. Both muscles of TSCmKO are more oxidative. Scale bar = 50 μm.

We have previously shown that deletion of *rptor* not only affects the immediate downstream targets of mTORC1, S6K and 4EBP, but also causes a strong increase in the phosphorylation of PKB/Akt [16]. As shown in Figure 3A, IRS1 levels were low in *soleus* muscle of TSCmKO mice compared to control (Figure 3A; Table 1). In addition, phosphorylation of PKB/Akt and of FoxO1/3 was substantially decreased in TSCmKO mice compared to controls (Figure 3A; Table 1). The same alterations in expression levels and phosphorylation of the examined proteins were detected in TA muscle of TSCmKO mice (data not shown). Consistent with the low phosphorylation levels of FoxO1a and FoxO3a, transcript levels of *atrogin-1/MAFbx* or *MuRF-1* were much higher in TA muscle of TSCmKO than in control mice (Figure 3B). Surprisingly, in *soleus* muscle, transcript levels of *atrogin-1/MAFbx* and *MuRF1* did not differ from controls (Figure 3C) despite the low levels of phosphorylation of PKB/Akt. These data argue that the differential expression of the two E3 ligases might be responsible for the selective hypertrophy in *soleus* muscle.

### Sustained activation of mTORC1 increases the oxidative capacity in all muscles

Additional factors that are regulated by mTORC1 [16,22,38] and have been implicated in the control of muscle size are the transcriptional coactivators PGC1α and PGC1β [39,40]. Moreover, PGC1α and PGC1β are major regulators of mitochondrial biogenesis [41]. To test whether deletion of *Tsc1* would also affect the PGC1 pathway and the oxidative capacity of skeletal muscle, we next compared expression of *Pgc1α* and *Pgc1β* in TA and *soleus* muscles of TSCmKO mice with littermate controls. Contrary to the expectation, transcript levels of *Pgc1α* were decreased in mutant muscles compared to controls (Figure 3D, E). The down-regulation of *Pgc1α* was more pronounced in *soleus* muscle, which expresses the highest level of PGC1α in wild-type mice [42]. In contrast, mRNA levels of *Pgc1β* were increased to about 150% in all examined muscles of TSCmKO mice (Figure 3D, E). In support of a direct regulation of *Pgc1β* transcripts by mTORC1, *Pgc1β* expression was diminished in RAmKO mice (*soleus* muscle in RAmKO mice: 73 ± 4.6%; control mice: 100 ± 10.3%; mean ± SEM; N ≥5; *P* <0.05). Hence, unlike expression of the E3 ubiquitin ligases *atrogin-1/MAFbx* and *MuRF1*, expression of *Pgc1α* and *Pgc1β* did not differ between TA and *soleus* muscles in TSCmKO mice. Overexpression and knockdown experiments of PGC1β in C2C12 myotubes indicate that expression of *Pgc1α* is tightly regulated by PGC1β (Figure 3F). Such counter-regulation between PGC1α and PGC1β has also been reported in other tissues [43]. Thus, the increased levels of *Pgc1β* transcripts in the TSCmKO mice likely suppress expression of *Pgc1α*. Interestingly, TSCmKO mice showed an increase in their capacity for oxidative phosphorylation in TA and *soleus* muscles as shown by stainings for NADH-TR (Figure 3G), succinate dehydrogenase (SDH; Additional file 1: Figure S4A, B) and cytochrome oxidase (COX; Additional file 1: Figure S4A, B). This increase was accompanied by a slight, although not significant, increase in the number of mitochondria as determined by qPCR of mitochondrial DNA (Additional file 1: Figure S4C). Taken together, these data suggest that PGC1β is responsible for the increased oxidative properties of skeletal muscle of TSCmKO mice.

### mTORC1 is required for muscle fiber hypertrophy

**Figure 4:**
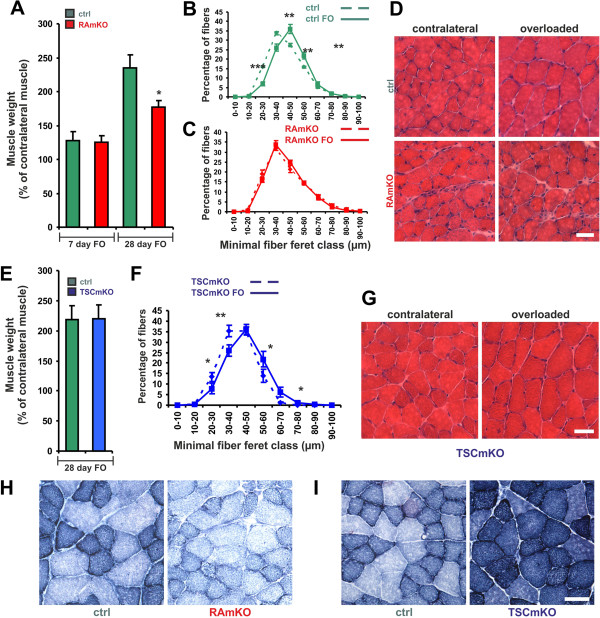
**Growth of muscle upon functional overloading.** (**A**) *Plantaris* muscles of control (ctrl) and RAmKO mice were functionally overloaded (FO) by ablation of the *soleus* and *gastrocnemius* muscles. Muscle weight of *plantaris* was measured after 7 or 28 days of FO and is expressed as the percentage of the weight of the contralateral, non-overloaded muscle (7 days FO: N ≥5 mice; 28 days FO: N ≥7 mice). (**B, C**) Fiber size distribution of the contralateral (dashed line) and FO (closed line) *plantaris* muscle of control (**B**) and RAmKO (**C**) mice after 28 days of FO (N = 7). (**D**) H&E staining of overloaded and contralateral *plantaris* muscles after FO for 28 days in control and RAmKO mice. (**E**) Muscle weight after 28 days of FO in control and TSCmKO mice (N = 5). (**F**) Fiber size distribution of non-overloaded, contralateral (dashed line) and over-loaded *plantaris* muscles (solid line) after 28 days of FO in TSCmKO mice (N = 5). (**G**) H&E staining of overloaded and contralateral *plantaris* muscles after 28 days of FO from TSCmKO mice. (**H, I**) NADH-TR staining of *plantaris* muscles after 28 days FO in mice with the indicated genotype. Scale bars (**D, G, H, I**) = 50 μm. Individual data points and bars of quantitative data represent mean ± SEM. *P*-values are ***P <0.001; **P <0.01; *P <0.05; Student’s *t*-test.

Because acute perturbation of mTORC1 function by knockdown experiments showed a strong effect on muscle size in experimental paradigms of HOR and denervation-induced atrophy (Figure 1), we next tested muscle plasticity in RAmKO and TSCmKO mice. We first used the synergist ablation/mechanical overload model, in which *gastrocnemius* and *soleus* muscles including their tendons are surgically removed, a procedure that results in the functional overloading (FO) of the remaining *plantaris* muscle [44-46]. Seven or 28 days after surgery, mice were euthanized and the *plantaris* muscle of the overloaded leg was compared with *plantaris* from the contralateral, sham-operated leg. In control mice, FO increased muscle weight after 7 days to 140% and to more than 200% after 28 days (Figure 4A). Muscle weight also increased in RAmKO mice, although the increase was significantly reduced compared to control animals after 28 days of FO (Figure 4A). However, and in contrast to control mice (Figures 4B and S5A), individual muscle fibers did not increase in size in RAmKO mice after 7 days (Additional file 1: Figure S5B) or after 28 days of FO (Figure 4C). H&E staining of the *plantaris* after 28 days of FO did not reveal differences between contralateral and overloaded RAmKO muscles (Figure 4D). In contrast to RAmKO mice, TSCmKO muscle responded to FO like control muscle (Figure 4E-G).

There is evidence that FO also causes some damage and muscle regeneration and that satellite and other cells outside the muscle’s basal lamina contribute to the weight increase [47,48]. As *HSA-Cre* is not expressed in non-muscle cells and satellite cells [24], we treated control mice with rapamycin during FO to eliminate mTORC1 function in all cells. This treatment abolished both the increase in weight and the shift in fiber size distribution (Additional file 1: Figure S5C), suggesting that mTORC1 expressed in non-muscle cells or in satellite cells might contribute to the increased weight of *plantaris* muscles in RAmKO mice after FO.

As FO induces a relative increase in the number of oxidative fibers [46], we also stained the overloaded *plantaris* from control and mutant mice by NADH-TR. As shown in Figure 4H, *plantaris* muscles remained largely non-oxidative in RAmKO mice, whereas in the overloaded *plantaris* of TSCmKO mice even the large myofibers remained highly oxidative (Figure 4I).

### *Soleus* and TA muscles of TSCmKO mice respond differently to denervation-induced atrophy

**Figure 5:**
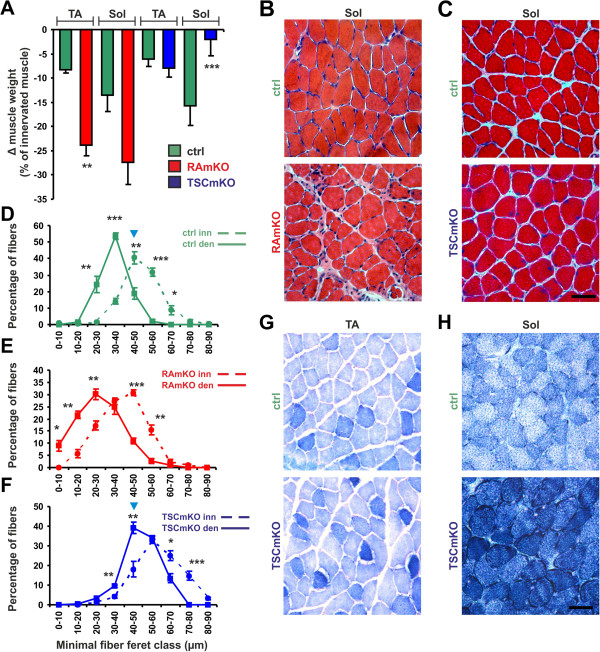
**Muscle atrophy induced by denervation.** (**A**) Loss (Δ) of muscle weight in *tibialis anterior* (TA) and *soleus* (Sol) muscles after six days of denervation using mice of the indicated genotype. Data are expressed as percentage of weight loss compared to the non-denervated contralateral muscle in the same mouse. N ≥4 mice for RAmKO and control littermates (ctrl); N ≥5 mice for TSCmKO and control littermates. (**B, C**) H&E staining of *soleus* muscle after six days of denervation in mice of the indicated genotype. (**D-F**) Fiber size distribution in *soleus* muscle after six days of denervation (solid line) and in the contralateral, non-denervated muscle (dashed line) of mice with the indicated genotype. Note that the most frequent fiber size in the denervated TSCmKO muscle is the same as that of innervated control muscle (blue arrowheads). N ≥4 for RAmKO and control littermates; N = 5 for TSCmKO and control littermates. (**G, H**) NADH-TR staining of TA and *soleus* muscles after six days of denervation in control and TSCmKO mice. Scale bars (B, C, G, H) = 50 μm. Quantification represent mean ± SEM. *P*-values are ***P <0.001; **P <0.01; *P <0.05 using the Student’s *t*-test.

To determine whether mTORC1 activation is sufficient to prevent atrophy, we next submitted TSCmKO muscle to denervation by cutting the sciatic nerve unilaterally and compared the muscles of the denervated and the contralateral (non-denervated) leg six days later. TA and *soleus* muscles of control mice lost 7% and 14% of their weight, respectively (Figure 5A). Importantly, the weight loss in both muscles was significantly higher in RAmKO mice (Figure 5A). In TSCmKO mice, the response to denervation differed between TA and *soleus* muscles. Whereas loss of weight in the TA was the same in TSCmKO and control mice, *soleus* muscles of TSCmKO mice were largely spared (Figure 5A). H&E staining of the denervated muscles and contralateral muscles did not reveal major structural changes in mutant mice (Figure 5B, C). In *soleus* muscles, the substantial weight loss upon denervation in control and RAmKO mice was mirrored by a shift in fiber size distribution. The leftward shift was seen in control mice (Figure 5D) and was even more pronounced in RAmKO mice (Figure 5E). In TSCmKO mice, muscle fiber size distribution also shifted slightly toward smaller size when compared to the hypertrophic, contralateral innervated *soleus* muscles (Figure 5F), but remained similar to innervated muscle from control mice. These results suggest that TA and *soleus* muscles differ in the response to mTORC1 activation under atrophy conditions and they suggest that the atrophy observed in the TSCmKO mice requires adaptive, long-term processes that are not induced by acute perturbation of mTORC1 signaling (see Figure 1). In both TSCmKO and control mice, the TA muscle showed a loss of oxidative capacity upon denervation (Figure 5G) whereas the *soleus* muscle of TSCmKO mice remained oxidative (Figure 5H).

### Feedback control of PKB/Akt is active during muscle atrophy

**Figure 6:**
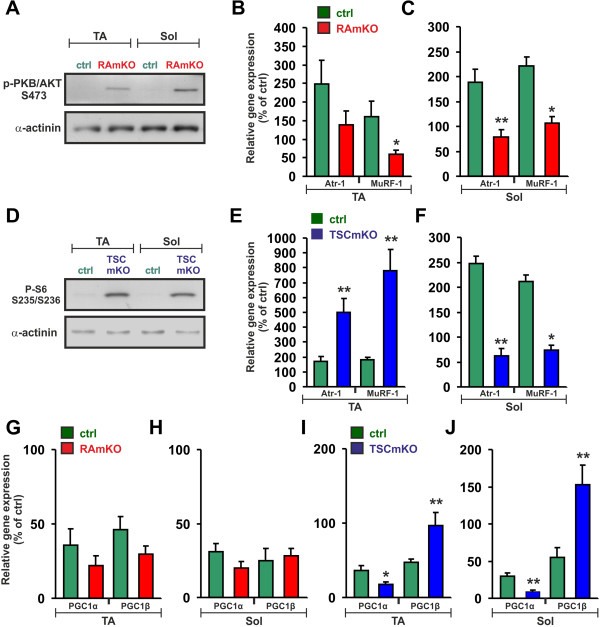
**Changes in mTORC1-dependent signaling in denervated muscles.** (**A, D**) Western blot analysis of *tibialis anterior* (TA) and *soleus* (Sol) muscles after six days of denervation using antibodies against PKB/Akt phosphorylated at Serine 473 (**A**) and S6 phosphorylated at Serines 235/236 (**D**). α-actinin was used as a loading control. (**B, C, E, F**) Relative mRNA levels of *atrogin-1/MAFbx* (Atr-1) and *MuRF1* as determined by qPCR in TA and *soleus* (Sol) muscles after six days of denervation. Note that expression of both E3 ligases is blunted in RAmKO mice (**B, C**), while this response is exaggerated in TA (**E**) but not in *soleus* muscles (**F**) of TSCmKO mice. (**G – J**) Relative mRNA levels of *Pgc1α* and *Pgc1β* in RAmKO (**G, H**) and TSCmKO (**I, J**) mice after six days of denervation. All values are normalized to the expression levels of the transcript measured in innervated muscle of control littermates (set to 100%). N ≥4 mice for TA and N ≥5 mice for *soleus* of each genotype. Values represent mean ± SEM. *P*-values are ***P <0.001; **P <0.01; *P <0.05; Student’s *t*-test.

The difference in the atrophy response between TA and *soleus* muscles indicated that the underlying signaling mechanisms might also differ in the two muscles. To examine this, we analyzed the changes in expression of the E3 ligases *atrogin-1/MAFbx* and *MURF1*, and the coactivators *Pgc1α* and *Pgc1β* in response to denervation. Denervation has been reported to activate mTORC1, most likely due to the increase in free amino acids [49]. However, in RAmKO mice phosphorylation of S6K, S6 and 4EBP remained low six days after denervation (Table 1 and data not shown) whereas phosphorylation at Serine 473 of PKB/Akt remained high in RAmKO mice (Figure 6A). In parallel to the activation state of PKB/Akt, denervation increased transcript levels of *atrogin-1/MAFbx* and *MuRF-1* in TA and *soleus* muscles of control mice but not of RAmKO mice (Figure 6B, C). The effect on the expression of the two E3 ubiquitin ligases was particularly striking in *soleus* muscles where their expression did not differ from innervated control muscles (Figure 6C). In TSCmKO mice, phosphorylated PKB/Akt was too low to be detected in denervated muscles (Table 1) but phosphorylation of S6 remained high (Figure 6D). Although phosphorylation of PKB/Akt was low in both TA and *soleus* muscles, transcript levels of *atrogin-1/MAFbx* and *MuRF-1* were increased in TA but were significantly lower in *soleus* compared to the denervated muscles from control mice (Figure 6E, F, Table 1).

The expression of the mTORC1 target PGC1α is also controlled by denervation [39]. In innervated *soleus* muscle of RAmKO mice, *Pgc1α* mRNA levels are less than 40% [16] and *Pgc1β* mRNA levels are approximately 70% of control muscle. In denervated TA and *soleus* muscles of control mice, expression of *Pgc1α* and *Pgc1β* was lower than in innervated muscle (Figure 6G, H). Similarly, denervation lowered the levels of both transcriptional co-activators in RAmKO mice although the significant difference to control mice was lost (Figure 6G, H). In contrast, expression of *Pgc1α* and *Pgc1β* was very different in TSCmKO mice. While *Pgc1α* mRNA levels were decreased upon denervation both in TA and *soleus* muscles, *Pgc1β* was significantly increased in both muscles (Figure 6I, J). Taken together, our results show that atrophy is accelerated in RAmKO mice despite low levels of *atrogin-1/MAFbx* and *MuRF1*. Conversely, the sparing of *soleus* muscles from denervation-induced atrophy in TSCmKO mice could be based on the low levels of the two E3 ubiquitin ligases in this particular muscle. In contrast, the relative levels of *Pgc1α* and *Pgc1β* did not differ between TA and soleus muscles upon denervation and are thus unlikely contributors to the differential response.

### Genetic inactivation of mTORC1 reverses the phenotype of TSCmKO mice

**Figure 7:**
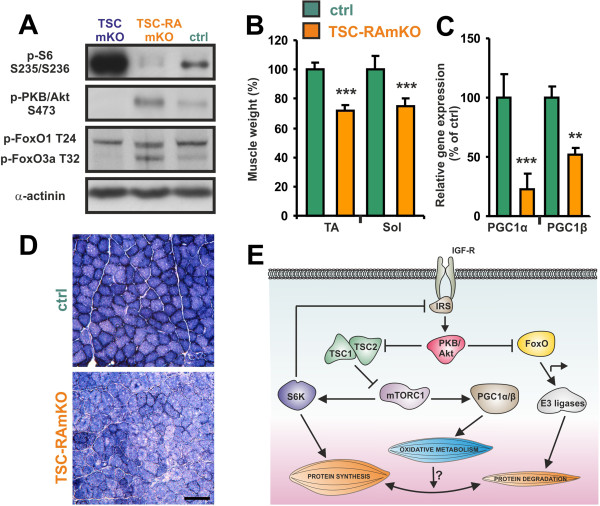
**TSC1-raptor double knockouts resemble RAmKO mice.** (**A**) Western blot analysis of *soleus* muscles of TSCmKO, TSC-RAmKO and control (ctrl) mice using antibodies directed against the proteins indicated. An equal amount of protein was loaded in each lane. Loading control was α-actinin. (**B**) Muscle weight of the *tibialis anterior* (TA) and *soleus* (Sol) muscles of TSC-RAmKO and control mice. Muscle weight was first normalized to the body weight and is expressed as percentage of the weight of the same muscle from control mice (N = 3 mice for each genotype). (**C**) Relative mRNA expression of *Pgc1α* and *Pgc1β* in *soleus* muscle of TSC-RAmKO and ctrl mice. Values obtained in control mice were set to 100% (N = 3 mice). Bars in B and C represent mean ± SEM. *P*-values are ***P <0.001; **P <0.01; *P <0.05. (**D**) NADH-TR staining of *soleus* muscle from TSC-RAmKO and control mice. Scale bar = 100 μm. (**E**) Schematic drawing of the major signaling pathways regulated by mTORC1 and their influence on protein synthesis and degradation.

While the inhibitory function of TSC1/2 onto mTORC1 is well established, there is evidence that this protein complex can also regulate mTORC2 [50,51]. To test whether any of the effects observed in TSCmKO mice would be maintained in RAmKO mice, we generated double knockout mice (termed TSC-RAmKO). First, we examined phosphorylation of known mTORC1 and mTORC2 substrates. As shown in Figure 7A, the mTORC1 substrate S6K and S6 were not phosphorylated in TSC-RAmKO mice and phosphorylation of PKB/Akt at Serine 473 was increased compared to control mice. In addition, similar to RAmKO mice, the PKB/Akt target FoxO3a was hyperphosphorylated. The weight of all muscles including TA and *soleus* was lower in TSC-RAmKO mice than in controls (Figure 7B). Moreover, transcript levels of both *Pgc1α* and *Pgc1β* were lower in *soleus* muscle (Figure 7C) and its oxidative capacity was decreased (Figure 7D). Finally, the TSC-RAmKO mice developed the same pathology as the RAmKO mice and they eventually died at the age of four to six months (data not shown). Thus, all the hallmarks of RAmKO mice are present in the double mutants, indicating that TSC acts mainly via mTORC1 in skeletal muscle.

## Discussion

Here we describe the phenotype of mice in which mTORC1 is constitutively active in skeletal muscle (TSCmKO) and compare it to mice with inactivated mTORC1 signaling (RAmKO). While the oxidative changes in TSCmKO mice were largely the opposite of those observed in RAmKO mice and affected all examined muscles, the effect of mTORC1 activation on muscle size was unexpected as all muscles except *soleus* muscles were slightly but significantly smaller. Thus, our work highlights the existence of several feed-forward or auto-inhibitory loops that allow fine-tuning of the signaling networks involved in the control of muscle mass (Figure 7E).

Based on the current concepts, mTORC1 activation should result in an increase in muscle mass and muscle fiber size. This view is based on the findings that activation of the mTORC1 upstream components PKB/Akt or IGF-1 receptor causes an increase in muscle mass [5,10,11,52,53] and that this increase is rapamycin-sensitive [11,53]. Moreover, overexpression of Rheb in single muscle fibers by electroporation leads to hypertrophy of the transfected fibers [20] and whole body knockout of the mTORC1 target S6K1 results in smaller muscle fibers [21]. Consistent with these experiments, acute knockdown of TSC1/2 by shRNA resulted in slightly bigger muscle fibers in *soleus* or TA muscles, confirming that transient activation of the mTORC1 pathway is sufficient to induce muscle fiber growth. However, under conditions of prolonged activation of mTORC1 in TSCmKO mice, all muscles examined, with the exception of *soleus*, were smaller than in control mice. As mTORC1 targets are activated and protein synthesis in EDL muscle of TSCmKO mice is increased, the atrophy induced by chronic mTORC1 activation is likely related to the feedback inhibition of activated S6K onto IRS1, which in turn, decreases activation of PKB/Akt. This tight feedback control of S6K on IRS1-PKB/Akt was also observed in mice deficient for raptor or mTOR in some tissues including skeletal and heart muscle [16,54-56] but not in others [57]. Similarly, deletion of TSC1 strongly decreases activation of PKB/Akt in cultured mouse embryonic fibroblasts [23], whereas it does not at all affect PKB/Akt phosphorylation in some tissues [58,59]. These data indicate that the feedback control of S6K depends on the cellular context and our data now show that this feedback is particularly strong in skeletal muscle.

Consistent with decreased inhibition of FoxO transcription factors by PKB/Akt, TA muscle from TSCmKO mice express high levels of *MuRF1* and *atrogin-1/MAFbx*, involved in protein degradation through the proteasome [7,8]. Hence, the atrophy observed in muscles of the TSCmKO mice is likely caused by the prevalence of the FoxO pathway over mTORC1 activation. This differs from the muscle hypertrophy observed using the transient, partial activation of mTORC1 with shRNA electroporation. Thus, the atrophy response caused by the sustained, saturated mTORC1 activation by genetic *Tsc1* deletion may unveil a long-term adaptation of the FoxO pathway. Consistently, transient overexpression of Rheb does not seem to affect PKB/Akt phosphorylation [20], further supporting the idea that muscle atrophy in TSCmKO mice is related to the indirect PKB/Akt-dependent activation of FoxO pathways.

Importantly, contrasting with the atrophic phenotype of most muscles, sustained activation of mTORC1 leads to increased mass of *soleus* muscle in TSCmKO mice. Although PKB/Akt was similarly inhibited in *soleus* and TA muscles, expression of *MuRF1* and *atrogin-1/MAFbx* was not increased in *soleus* muscle*,* indicating that an additional regulatory mechanism suppresses their expression, thereby overruling the regulation by PKB/Akt. This differential regulation of *MuRF1* and *atrogin-1/MAFbx* expression did not seem to be mediated by PGC1α, previously identified as a negative regulator of FoxO [39], because there was no significant difference in PGC1α/β expression between *soleus* and TA muscles from TSCmKO mice.

With different atrophy and hypertrophy paradigms, we also demonstrate that mTORC1 plays a critical and complex role in muscle plasticity. Using shRNA electroporation, we show that transient activation of mTORC1 is sufficient to limit denervation-induced atrophy and to enhance fiber hypertrophy upon re-innervation. Similarly, TSCmKO mice display atrophy resistance to denervation in *soleus* muscle, which shows only moderate expression of the E3 ubiquitin ligases *MuRF1* and *atrogin-1/MAFbx*. By contrast, long-term activation of mTORC1 did not protect TA muscle from atrophy and did not exacerbate the hypertrophy response to overloading of *plantaris* muscle. These results indicate that the increased protein synthesis by mTORC1 hyperactivation is not sufficient to maintain muscle mass in cases where the FoxO-MuRF1-atrogin-1/MAFbx axis is active due to the absence of PKB/Akt signaling. Importantly, both transient and long-term inactivation of mTORC1 increased denervation-induced atrophy and prevented muscle growth associated with re-innervation or overloading, indicating that increased protein synthesis is required even when the catabolic proteasomal activity is reduced. Thus, our results provide genetic evidence that muscle growth requires mTORC1.

In our previous work, we demonstrated that raptor-deficient skeletal muscles show a strongly decreased oxidative capacity due to changes in mitochondrial function [16]. This loss of oxidative capacity correlated with a substantial decrease in the transcript levels of *Pgc1α*, consistent with the direct regulation of *Pgc1α* expression by mTOR [38], and could be restored by transgenic expression of PGC1α [22]. Contrary to the expectations and the effect of mTORC1 activation in embryonic fibroblasts [38], all examined muscles of TSCmKO mice showed a decreased expression of *Pgc1α* but increased levels of *Pgc1β*. Thus, the increase in the oxidative capacity in TSCmKO mice may be mediated by PGC1β. Indeed, PGC1β has also been shown to be sufficient to increase oxidative capacity in skeletal muscle despite the concomitant reduction in PGC1α expression [60]. Moreover, depletion of both PGC1α and PGC1β results in much more severe loss of oxidative capacity than depletion of either protein alone [61]. The reason for the unexpected down-regulation of *Pgc1α* transcripts in TSCmKO mice might be the counter-regulation of PGC1α and PGC1β. We show here that overexpression of PGC1β in C2C12 myotubes results in a strong suppression of the endogenous *Pgc1α* expression and, conversely, *Pgc1β* knockdown leads to increased expression of *Pgc1α* transcripts. These data indicate that the total amount of both PGC1 co-activators is tightly controlled in skeletal muscle.

## Conclusions

Our study provides new functional insights into the molecular mechanism of muscle atrophy and hypertrophy. The data demonstrate that mTORC1 modulation downstream of PKB/Akt is subject to biological robustness. A fine-tuned feedback loop controlled by the anabolic mTORC1 pathway mediates crosstalk to E3 ubiquitin ligase system that increases protein degradation and thus compensates for imbalance. However, this feedback system fails to fully re-establish muscle homeostasis, leading to prevalence of either an anabolic or a catabolic net response. Our observations emphasize that muscle growth requires both activated PKB/Akt and mTORC1 in parallel, and they provide a new rationale for the development of pharmacologic agents that target this system.

## Abbreviations

4EBP: eIF-4E-binding protein; BSA: Bovine serum albumin; CSA: Cross-section area; FoxO: Forkhead box O; FO: Functional overloading; H&E: Hematoxylin & eosin; HOR: Hypertrophy on recovery; HSA: Human skeletal actin; IGF1: Insulin-like growth factor-1; i.p: Intraperitoneal; mTORC1: Mammalian target of rapamycin complex 1; NADH-TR: Nicotinamide adenine dinucleotide hydrogen-tetrazolium reductase; NLS-GFP: Nuclear-localized GFP; PBS: Phosphate-buffered saline; PFA: Paraformaldehyde; PI3K: Phosphatidylinositol 3-kinase; PKB: Protein kinase B (also called Akt); PGC-1: Peroxisome proliferator-activated receptor gamma coactivator 1; Raptor: Regulatory-associated protein of mTOR; RAmKO: Raptor muscle knockout; Rheb: Ras homolog enriched in brain; Rictor: Rapamycin-insensitive companion of mTOR; S6K: p70/S6 kinase; SUnSET: Surface sensing of translation; TA: Tibialis anterior; TBST: Tris-buffered saline with 0.1% Tween 20; TSC: Tuberous sclerosis complex; TSCmKO: TSC muscle knockout

## Competing interests

The authors declare they have no competing interests.

## Authors’ contributions

CFB, SL and MAR conceived and designed the study. CFB and SL performed most of the experiments and analyzed the data. KR, PC, MG and SS conducted some of the experiments and CH, LAT and MNH provided scientific input. CFB, SL, PC and MAR wrote the manuscript. All authors read and approved the final manuscript.

## Supplementary Material

Additional file 1 **Contains supplemental figures S1 to S5 and supplemental Table S1.** See text and additional file 1 for more details. Click here for file
