# Supplementary material for: Differential response of skeletal muscles to mTORC1 signaling during atrophy and hypertrophy
Source: Skelet Muscle. 2013 Mar 6;3:6. doi: 10.1186/2044-5040-3-6 (PMC3622636; doi:10.1186/2044-5040-3-6)
Supplement: Additional file 1 — Contains supplemental figures S1 to S5 and supplemental Table S1. See text and additional file 1 for more details. [file 2044-5040-3-6-S1.pdf]

Supplemental Figures and Table

Differential response of skeletal muscles to mTORC1 signaling during atrophy and hypertrophy

C. F. Bentzinger, S. Lin, K. Romanino, P. Castets, M. Guridi, S. Summermatter, C. Handschin, L. A. Tintignac, M. N. Hall and M. A. Rüegg

5 Supplemental Figures

1 Supplemental Table

Figure S1

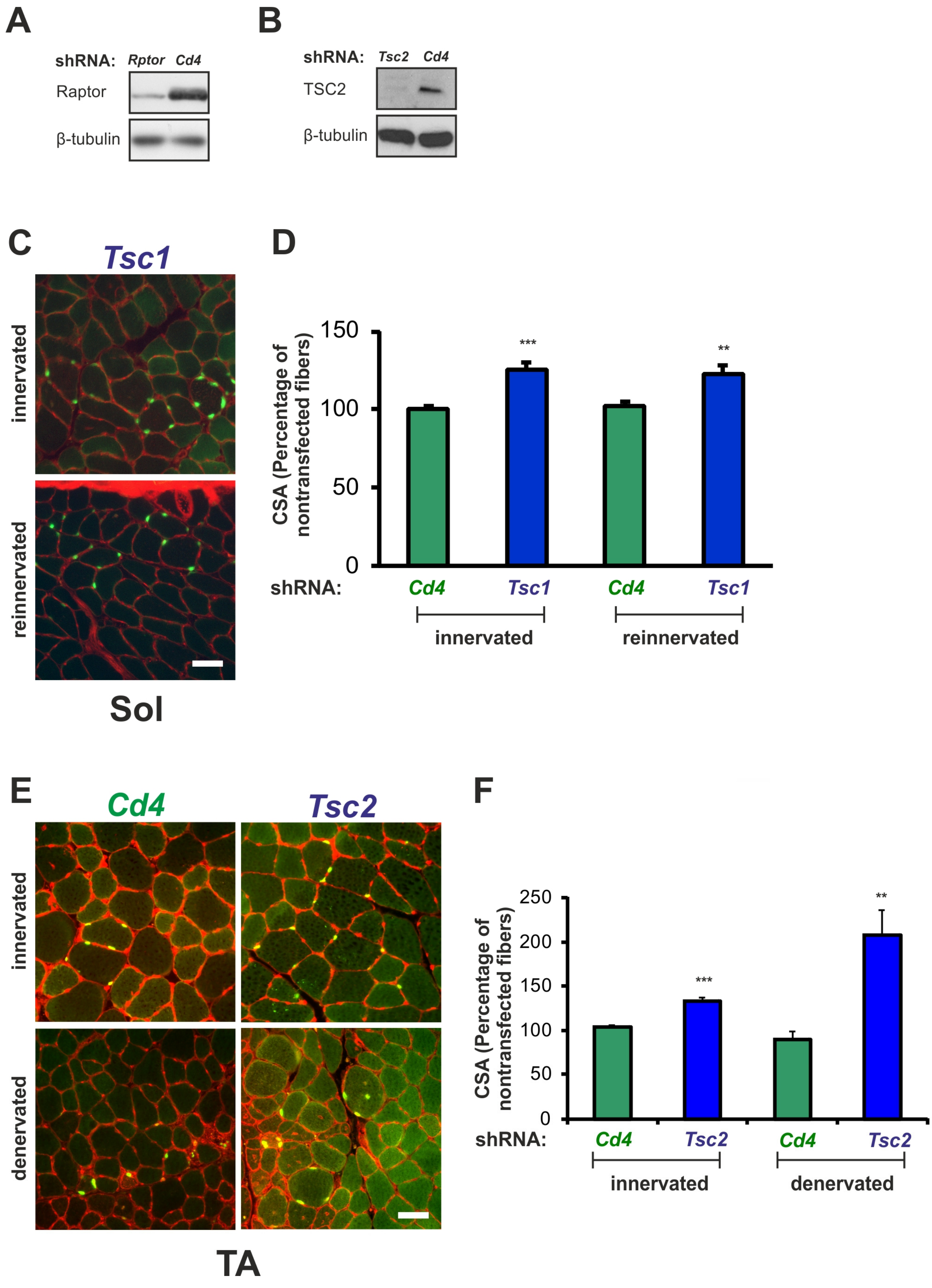
A
shRNA: Rptor Cd4
Raptor
β-tubulin
B
shRNA: Tsc2 Cd4
TSC2
β-tubulin
C
Tsc1
innervated
reinnervated
Sol
D
CSA (Percentage of nontransfected fibers)
150
100
50
0
***
**
shRNA:
Cd4
Tsc1
Cd4
Tsc1
innervated
reinnervated
E
Cd4
Tsc2
innervated
denervated
TA
F
CSA (Percentage of nontransfected fibers)
250
200
150
100
50
0
***
**
shRNA:
Cd4
Tsc2
Cd4
Tsc2
innervated
denervated

Figure S1. **Knockdown of TSC1 or TSC2 in results in muscle hypertrophy**

(**A**) Western blot analysis of COS cells that were co-transfected with an expression construct encoding raptor and a construct encoding shRNA against *rptor* or *Cd4*. (**B**) Myoblasts infected with an adenovirus encoding shRNA against *Tsc2* or *Cd4*. Knockdown of expressed protein was determined using indicated antibodies. Equal amounts of protein were loaded as indicated by the expression of β-tubulin. (**C-F**) *Soleus* (**C** and **D**) and TA (**E** and **F**) muscles were electroporated with cDNA constructs encoding shRNA against *Tsc1*, *Tsc2* or *Cd4*. After four to six weeks, muscle fiber size was determined by staining mid-belly cross-sections with Alexa-594-labeled wheat germ agglutinin (red). Electroporated muscle fibers were identified by the expression of nuclear-localized GFP (green). Muscles analyzed were innervated, reinnervated or denervated as indicated. Scale bar = 50 μm. (**D** and **F**) Quantifications of cross-sectional area (CSA) of muscle fibers in each paradigm are given relative to CSA of neighboring, non-electroporated fibers. Bars represent mean ± SEM (N = 3 mice). P-values are ***p < 0.001; **p < 0.01; p-values were determined by student's t-test.

Figure S2

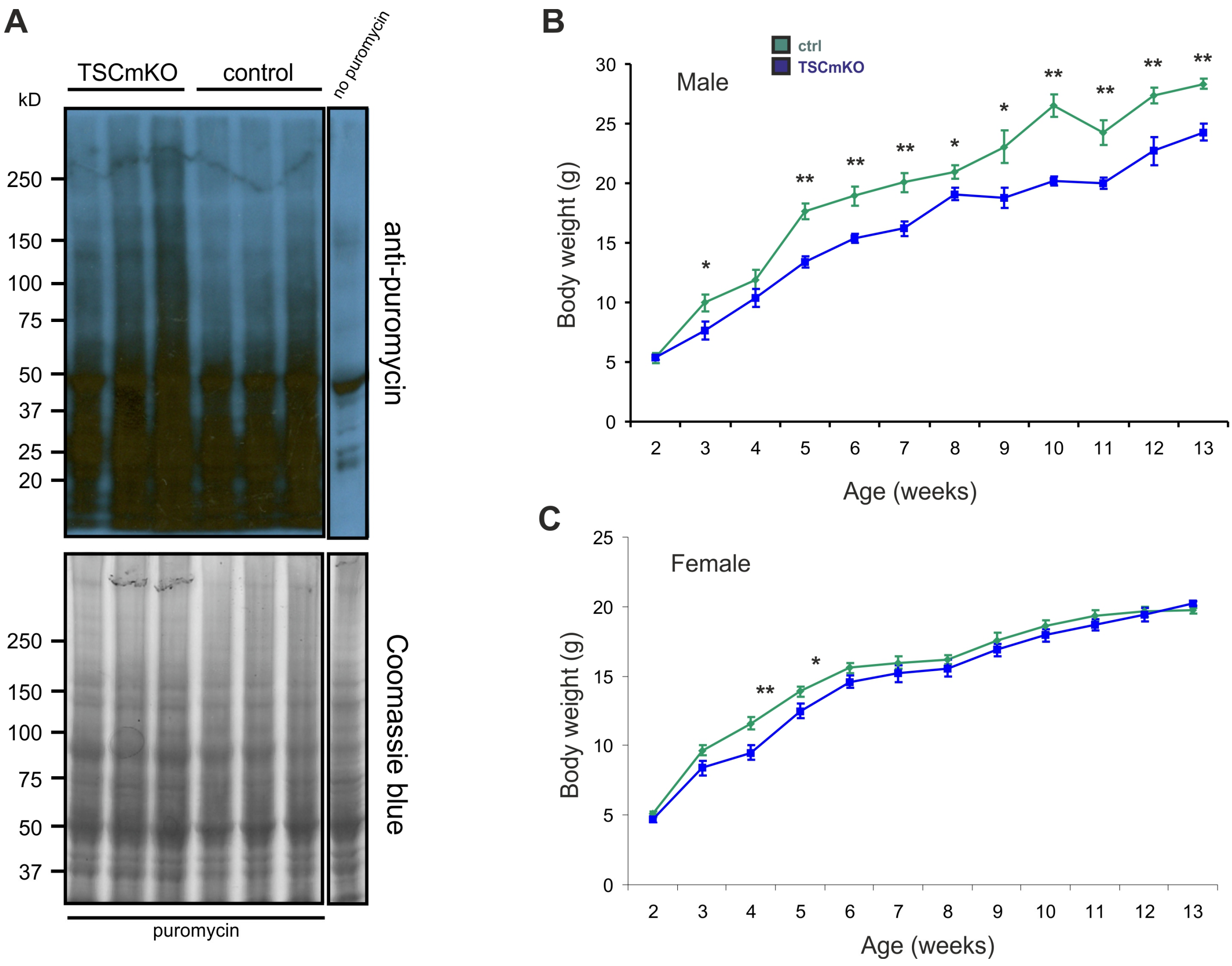

Figure S2. **Increased protein synthesis and lower weight gain in TSCmKO mice**
(**A**) Western blot analysis of puromycin incorporation in total protein extract of EDL muscle from 60 day-old TSCmKO and control mice injected with puromycin. Sham-injected mice show no specific bands demonstrating the specificity of the anti-puromycin antibody (rightmost lane). Coomassie Blue staining of the gel (bottom part) demonstrates equal protein loading. (**B, C**) Body weight of male (**B**) and female (**C**) TSCmKO and control mice (N = 4 - 35 mice for each genotype and gender). Values represent mean ± SEM. P-values are **p < 0.01; *p < 0.05 as determined by student's t-test.

Figure S3

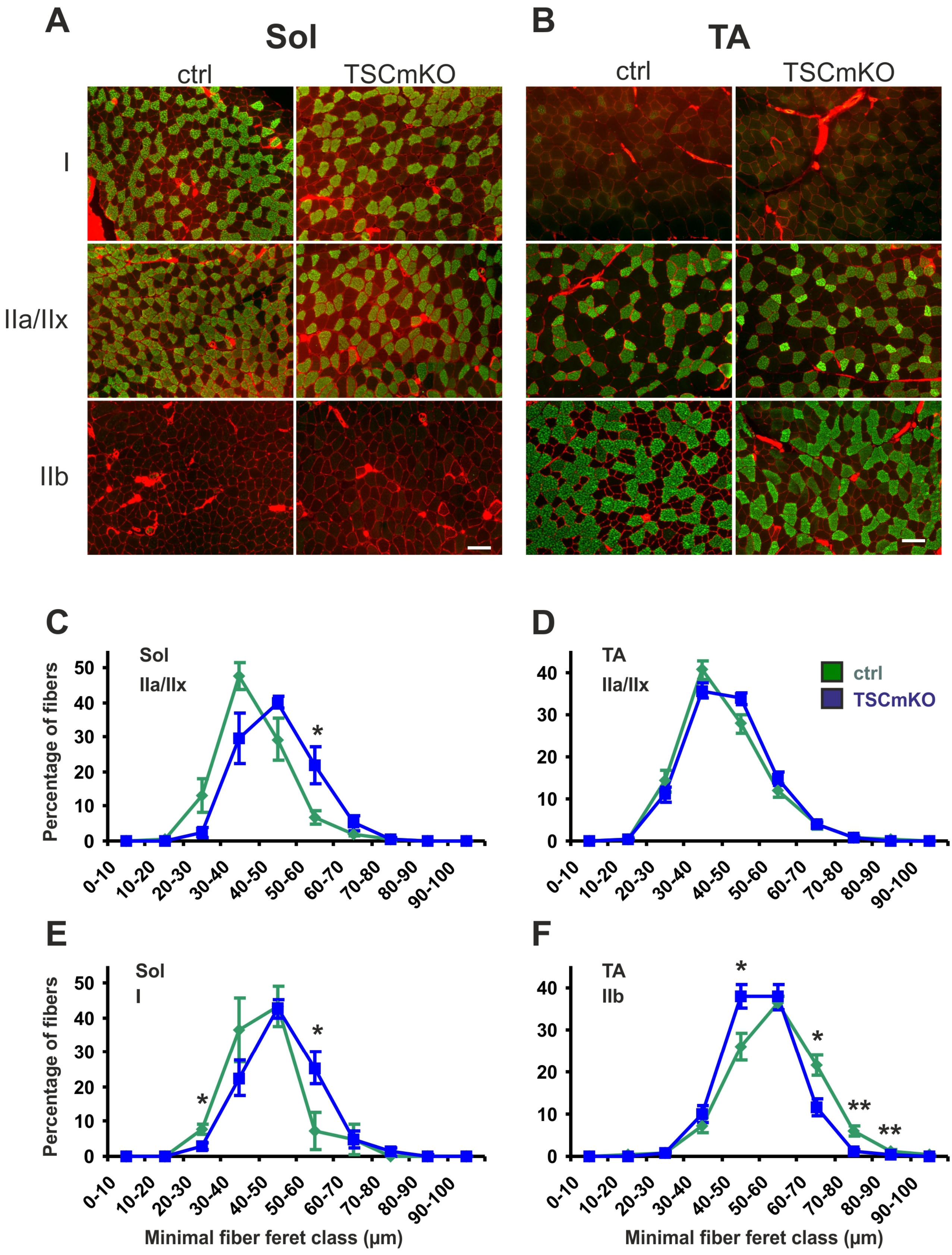

Figure S3. **Quantification of muscle fiber size of individual fiber types**

(**A, B**) Representative images of *soleus* (**A**) and TA (**B**) muscles stained for fiber subtype as indicated. (**C** - **F**) Fiber size distribution of indicated fiber subtype of *soleus* (**C** and **E**) and TA (**D** and **F**) muscles. Note that all the fibers in *soleus* muscle are bigger in TSCmKO than in control mice. In contrast, type IIb fibers in TA muscle are smaller in TSCmKO than in control mice. N = 4 mice in each genotype. Values represent mean ± SEM. P-values are $^{**}p < 0.01$; $^{*}p < 0.05$; determined by student's t-test.

Figure S4

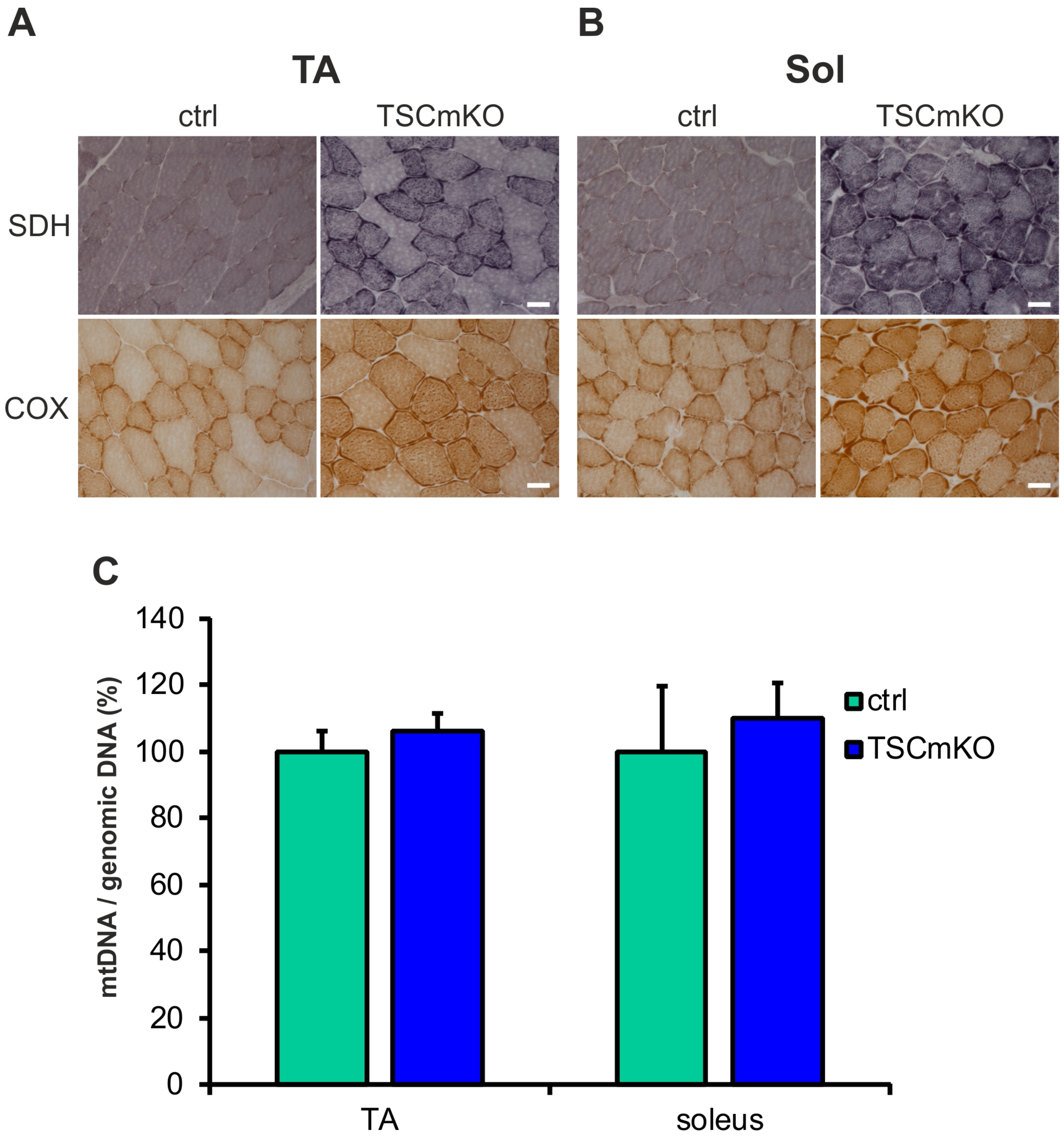

Figure S4. **Muscles of TSCmKO mice are more oxidative**

(**A** and **B**) SDH and COX staining of TA (**A**) and *soleus* (**B**) muscles of 90 day-old control (ctrl) and TSCmKO mice. Both muscles of TSCmKO mice are more oxidative (N = 4 mice). Scale bar = 50 μm. (**C**) Quantification of the copy number of mitochondrial DNA (mtDNA) in TA and *soleus* muscle of 90 day-old ctrl and TSCmKO mice (N = 3 mice) relative to the amount of genomic DNA. Data represent mean ± SD; N = 4 ctrl and N = 3 TSCmKO mice.

Figure S5

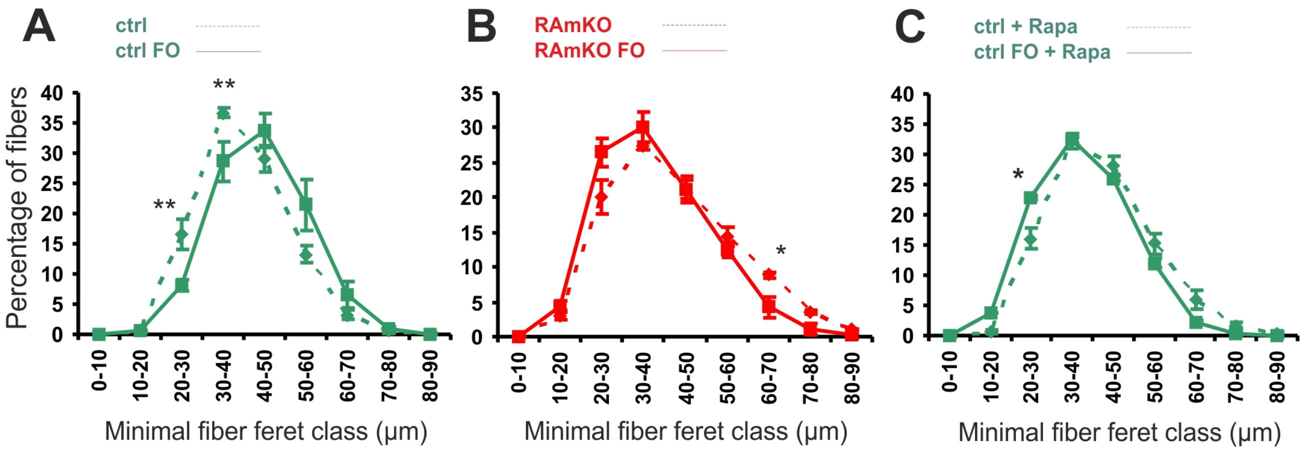

Figure S5. **Increase in muscle fiber size requires mTORC1**

(**A, B**) Fiber size distribution of *plantaris* muscle, which was functionally overloaded (FO) for 7 days (solid lines) and of non-overloaded, contralateral muscle (dashed lines). While the fiber size distribution is clearly shifted toward bigger fibers in control mice (**A**), no shift was seen in RAmKO mice (**B**). (**C**) Daily application of rapamycin prevented muscle fiber growth in *plantaris* muscle of control mice by overloading for 28 days (N = 4 mice in all experiments). Values represent mean ± SEM. P-values are **p < 0.01; *p < 0.05; student's t-test was used to calculate p-values.

Table S1

			soleus			TA		
			**ctrl**	**TSCmKO**	***p*-value**	**ctrl**	**TSCmKO**	***p*-value**
	**total**	fiber number	854 ± 50	848 ± 35	0.956	2898 ± 129	2886 ± 200	0.862
		fiber feret (µm)	40.01 ± 1.59	45.67 ± 1.55	0.044	47.42 ± 0.82	47.05 ± 0.99	0.784
**Fiber type**	**I**	fiber number (%)	38.53 ± 1.62	41.95 ± 3.95	0.453	n.d.	n.d.	n.d.
		fiber feret (µm)	41.06 ± 1.82	46.02 ± 1.47	0.078	n.d.	n.d.	n.d.
	**IIa**	fiber number (%)	55.67 ± 3.27	59.91 ± 1.23	0.270	12.14 ± 3.67	10.60 ± 1.25	0.705
		fiber feret (µm)	37.68 ± 1.62	44.62 ± 1.92	0.033	34.37 ± 1.96	35.19 ± 0.67	0.705
	**IIx**	fiber number (%)	8.01 ± 2.26	6.89 ± 1.43	0.690	44.66 ± 3.61	35.69 ± 1.50	0.062
		fiber feret (µm)	44.96 ± 2.58	46.58 ± 1.79	0.627	41.75 ± 0.96	43.53 ± 0.96	0.238
	**IIb**	fiber number (%)	n.d.	n.d.	n.d.	53.29 ± 2.93	66.70 ± 2.66	0.015
		fiber feret (µm)	n.d.	n.d.	n.d.	54.48 ± 1.20	50.29 ± 1.01	0.037

Table S1. **Characterization of muscles from TSCmKO mice**

The number of fibers and their mean feret were determined according to fiber types, in *soleus* and TA muscles from 90 day-old TSCmKO and control (ctrl) mice. n.d.: not detected. Values are mean ± SEM; p-values were determined by student's t-test.
